# Mice with humanized immune system as novel models to study HIV-associated pulmonary hypertension

**DOI:** 10.3389/fimmu.2022.936164

**Published:** 2022-08-05

**Authors:** Valerie J. Rodriguez-Irizarry, Alina C. Schneider, Daniel Ahle, Justin M. Smith, Edu B. Suarez-Martinez, Ethan A. Salazar, Brianyell McDaniel Mims, Fahmida Rasha, Hanna Moussa, Naima Moustaïd-Moussa, Kevin Pruitt, Marcelo Fonseca, Mauricio Henriquez, Matthias A. Clauss, Matthew B. Grisham, Sharilyn Almodovar

**Affiliations:** ^1^ Department of Immunology and Molecular Microbiology, Texas Tech University Health Sciences Center, Lubbock, TX, United States; ^2^ Department of Biology, University of Puerto Rico in Ponce, Ponce, PR, United States; ^3^ Division of Pulmonary Sciences and Critical Care Medicine, University of Colorado Anschutz Medical Campus, Aurora, CO, United States; ^4^ Department of Mechanical Engineering, Texas Tech University, Lubbock, TX, United States; ^5^ Department of Nutritional Sciences, Texas Tech University, Lubbock, TX, United States; ^6^ Program of Physiology and Biophysics, University of Chile, Santiago, Chile; ^7^ Pulmonary, Critical Care, Sleep and Occupational Medicine, Indiana University, Indianapolis, IN, United States

**Keywords:** HIV, HIV-associated pulmonary hypertension, HIV-PH, HIV-PH Pulmonary hypertension, EMAP II, hypoxia, SU5416, Humanized mice

## Abstract

People living with HIV and who receive antiretroviral therapy have a significantly improved lifespan, compared to the early days without therapy. Unfortunately, persisting viral replication in the lungs sustains chronic inflammation, which may cause pulmonary vascular dysfunction and ultimate life-threatening Pulmonary Hypertension (PH). The mechanisms involved in the progression of HIV and PH remain unclear. The study of HIV-PH is limited due to the lack of tractable animal models that recapitulate infection and pathobiological aspects of PH. On one hand, mice with humanized immune systems (hu-mice) are highly relevant to HIV research but their suitability for HIV-PH research deserves investigation. On another hand, the Hypoxia-Sugen is a well-established model for experimental PH that combines hypoxia with the VEGF antagonist SU5416. To test the suitability of hu-mice, we combined HIV with either SU5416 or hypoxia. Using right heart catheterization, we found that combining HIV+SU5416 exacerbated PH. HIV infection increases human pro-inflammatory cytokines in the lungs, compared to uninfected mice. Histopathological examinations showed pulmonary vascular inflammation with arterial muscularization in HIV-PH. We also found an increase in endothelial-monocyte activating polypeptide II (EMAP II) when combining HIV+SU5416. Therefore, combinations of HIV with SU5416 or hypoxia recapitulate PH in hu-mice, creating well-suited models for infectious mechanistic pulmonary vascular research in small animals.

## Introduction

People living with Human Immunodeficiency Virus infection suppressed by antiretroviral therapy (ART) have experienced a tremendous improvement in life expectancy. Still, there is a devastating gap of 1-2 decades of lifespan between HIV-infected and uninfected individuals that affect their quality of life (1, 2). ART does not cure HIV infection and patients struggle with infectious and non-infectious complications of chronic HIV infection. In patients aging with HIV, low-level viral replication persists within tissues like the lungs, which imposes important challenges to viral eradication after long-term ART and serious co-morbidities due to chronic inflammation (3–9). Pulmonary vascular cell dysfunction precedes vascular remodeling, which may result in life-threatening Pulmonary Hypertension (PH) depending on the type and degree of the injury at the molecular and cellular levels. PH is characterized by significant vascular inflammation, progressive increase of pulmonary vascular resistance, and obliterative vascular remodeling resulting in right ventricular dysfunction, heart failure, and premature death (10–13). People living with HIV face an approximate 2000-fold increase in developing PH, even in the era of ART (14–16). Patients with viral loads > 500 copies/mL and CD4^+^ cell counts < 200 cells/uL have a higher prevalence of increased pulmonary artery systolic pressures ≥40 mmHg and mortality than uninfected patients (17). While the presence of HIV seems a common denominator in HIV-PH (18, 19), the mechanisms of how HIV causes or contributes to PH remain undeciphered. The use of animal models in both HIV and PH research areas has filled several gaps in knowledge regarding infectious HIV and/or HIV proteins as pathogenic insults to the lungs.

In HIV research, non-human primates have historically been used for studies with the simian counterpart of HIV (SIV) or SIV/HIV (SHIV) chimeric viruses, which have successfully advanced the field of HIV/SIV pathogenesis over decades (20–25). Importantly, the inclusion of transgenic rats and mice in HIV/AIDS research have facilitated this endeavor using the expression of HIV proteins in the absence of infection (26, 27). Therefore, the impact of specific HIV proteins on several molecular and organ systems can be studied in detail in small animals, with minimal occupational hazards, significantly lower costs, and wide availability, compared to infected primates.

A major revolution in the study of the immunopathology of HIV infection in small animals was the humanization of the mouse immune system because they provide immune cells susceptible to HIV infection (28–30). The successful and long-term engraftment of human lymphocytes or hematopoietic stem cells (HSCs) arose from studies demonstrating that deletion or inactivation of the IL-2 receptor common gamma chain (IL-2rγ) in lymphopenic mice produced animals lacking T, B and NK cells (31). Currently, there are several stocks of lymphopenic-IL2rγ deficient (LP-IL2rγ^-/-^) mice that are used to engraft human T cells or HSCs. One of the most commonly used stocks is the non-obese diabetic/SCID (NOD/SCID)-IL2rγ^-/-^ or NSG mouse (32, 33). NSG mice or a comparable LP-IL2rγ^-/-^ strain has been used extensively to promote the engraftment, differentiation, and expansion of human CD3^+^ HSCs. To do this, sub-lethally-irradiated NSG mice are engrafted with small fragments of human fetal liver and thymus under the kidney capsule followed by intravenous injection of donor-matched HSCs (34). Using this protocol, investigators have observed the presence of human T and B cells, NK cells, DCs, monocytes, and macrophages in the peripheral blood at 4-8 weeks post-engraftment (35). This model, called the bone marrow-liver-thymus immune humanized mouse or hu-BLT model, creates a thymic organoid where HSC-derived T cell progenitors interact with human leukocyte antigens (HLA) within the thymus tissue to produce HLA Class I- and Class II-restricted T cells. Hu-mice support multi-organ HIV infection (36), undergo HIV-induced CD4^+^ T cell depletion and exhibit latency when treated with antiretroviral therapy (36–42). Therefore, hu-mice have significantly hastened the HIV immunopathogenesis field (29, 30) due to its high translatability (43).

In the field of Pulmonary Hypertension, several animal models including mice, rats, sheep, cows, and monkeys have helped describe and understand molecular mechanisms in the development and progression of PH (44–46). The understanding of the biphasic pathogenesis of PH is key in the assessment of the *in vivo* models available. Specifically, phase 1 consists of non-specific medial and adventitial thickening of the pulmonary arteries, followed by phase 2 with occlusive plexiform lesions (47). According to modern synchrotron-based micro-CT studies, plexiform lesions in PH are observed either in monopodial branches (pulmonary arteries that are not associated with the airways), between pulmonary arteries and larger airways, at the end of distal pulmonary arteries, or within pulmonary arteries where the lesion obstructs the blood flow, which gets naturally redirected to smaller lumina (recanalization) (48). Non-human Primates can recapitulate phase 2 obliterative angioproliferative lesions in the pulmonary vasculature, which makes them valuable in the field of PH research (49–54). Approaches for the induction of experimental PH vary broadly according to the goals of each study, particularly in small animals. Experimental models of PH have combined hypoxia with the use of a synthetic inhibitor of the vascular endothelial growth factor [VEGF] receptor-2 (Sugen 5416 or SU5416) to generate widely used models of PH in rats (55–58) and mice (59–61). Revealing studies by Oka et al. (56) demonstrated that SU5416-hypoxia-normoxia rats recapitulate the pulmonary arteriopathy seen in severe human PH, including two different patterns of complex lesions that represent both early-stage intraluminal plexiform lesions (stalk-like) and aneurism-like complex lesions characteristic of PH-associated congenital heart disease (62). These histopathological features were observed in rats treated with SU5416 and exposed to hypoxia for 3 weeks followed by normoxia for 10-11 weeks (56). Besides the PH models including hypoxia, longstanding models using monocrotaline (MCT) pyrrole alkaloid still dominate the preclinical PH research area because of its striking capacity to recapitulate PH in rats after a single injection (44, 63, 64). Interestingly, the variety of mechanisms by which experimental models lead to PH is as diverse as the disease in itself. For instance, the MCT PH rat model is widely characterized by medial hypertrophy in the pulmonary arteries (with main involvement of pulmonary artery smooth muscle cells) but does not exhibit obliterative endothelial cell pathology as portrayed in the SU5416/hypoxia model (64, 65).

Therefore, the multifaceted aspects in the development of PH are an intrinsic complexity in the research of HIV-PH, which requires animal models that can mimic both pathologies. In the context of HIV-PH, rhesus macaques infected with chimeric SHIV-nef showed for the first time, that the HIV Nef protein co-localizes with pulmonary artery endothelial cells within plexiform lesions (51, 66). Longitudinal studies in SIV-infected monkeys showed that pulmonary hemodynamic alterations are unrelated to viral load, sex, or CD4^+^ counts but rather correlated with inflammation and increased right ventricular and pulmonary arterial fibrosis. Additional PH models have also combined SIV infection and morphine as known pulmonary vascular insults in rhesus macaques (53).

In addition to primates, HIV transgenic rodents have successfully demonstrated that HIV proteins significantly increased pulmonary vascular resistance. Pulmonary arteriopathy response may be intensified by factors such as cocaine or hypoxia, leading to increased right ventricular systolic pressures, right ventricular hypertrophy, vessel muscularization, and pulmonary hypertension in rats (67–69). In addition, the combination of SU5416 + morphine in rats (70) successfully triggered pro-angiogenic signaling and vascular remodeling as hallmarks of PH. These models have provided mechanistic insights to help understand PH development and progression, including the novel role of RNA 216a as a novel repressor of bone morphogenetic protein receptor type 2 (BMPR2) translation in HIV transgenic rats (71). Transgenic HIV expression in mice demonstrated significant impairment in pH-sensitive potassium channels even in the absence of hemodynamic changes (72). While transgenic animals serve to rule in/out the roles of viral proteins in PH, these non-infectious models cannot mimic the initial infectious HIV disease and the pulmonary immune responses to viruses required to fully understand the pathobiology of HIV-PH at its bud.

HIV is a human-tropic virus that only infects human cells; hence, the use of humanized mice warrants fundamental considerations for the continued advancement of HIV-PH animal models. Moreover, given that previous studies in rodents have demonstrated the role of immune cells in PH (73, 74), it would be imperative to test this hypothesis in mice carrying an immune system that is a target of HIV. The presence of HIV proteins alone tends to decrease VEGF expression in SIV-infected macaques with pulmonary arteriopathy (53). Therefore, adopting the SU5416 approach from the PH animal models would be justifiable. Although hypoxic mice with humanized immune systems have proved useful for PH studies (75), the validity of HIV-infected humanized mice for pulmonary hemodynamic and mechanistic studies in HIV-PH has not been established.

Herein, we adapted the dual-hit concept from the well-established SU5416/hypoxia model of PH and used HIV-infected mice with humanized immune systems to test two novel models (HIV/SU5416 and HIV/hypoxia) that may be valuable in further HIV-PH studies.

## Materials and methods

### Humanization of the mouse immune system

**Figure 1:**
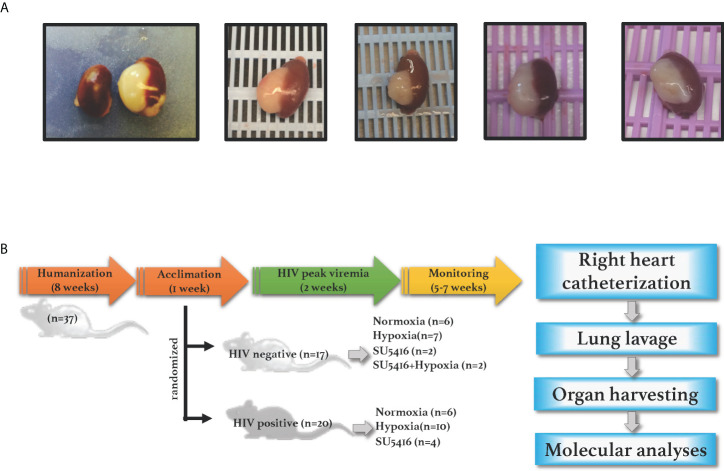
**(A)** Human spleen and liver organoids in the mouse kidney capsule. NSG BLT mice were engrafted with fetal liver, thymus and hematopoietic cells; human engraftments were confirmed by gross inspections of the mouse kidney capsules at euthanasia, where organoids develop in humanized mice. **(B)** Experimental timeline. NSG mice were engrafted with either human hematopoietic CD34+ cells (cord-blood derived) or with human liver, thymus, and hematopoietic stem cells (fetal-derived). After confirmation of human engraftments by flow cytometry, humanized mice were infected with HIV and either placed in hypoxia or injected with SU5416 to induce PAH. Animals were monitored for any signs of disease including graft-vs-host disease, labored breathing or wasting throughout the course of the study. Mice were subjected to open-chest right heart catheterizations under anesthesia; specimens were collected for further analyses.

NSG mice (NOD. Cg-*Prkdc^scid^ Il2rg^tm1Wjl^* (NSG, JAX stock number 005557) were purchased from The Jackson Laboratory and engrafted with either fetal tissues or CD34+ stem cells. BLT mice were engrafted with human fetal liver and thymus under the renal capsule, followed by injection with donor-matched liver hematopoietic stem cells (HSC) *via* tail vein, as per established protocols to produce the BLT mouse model (76, 77). The level of engraftment in the BLT mice was assessed by flow cytometry 12 weeks post-humanization by measuring CD45^+^ cells in peripheral blood by flow cytometry (data not shown); only the mice with > 70% human CD45^+^ cells in the periphery were used in this study. Engraftments were further confirmed by gross observations of the presence of human spleen and thymus organoids in the mouse kidney capsule at euthanasia **(** **Figure 1A** **).** Mice humanized with CD34^+^ stem cells were purchased from The Jackson Laboratory and showed a 31-48% level of human CD45^+^ cell engraftment.

All mice were monitored daily for the appearance of graft-vs-host disease (GvHD) symptoms, including weight loss, blepharitis, hunched posture, ruffled fur, hair loss, reduced mobility, and tachypnea. The presence of any of these phenotypes determined the time for humane euthanasia. All animals were housed in pathogen-free cages; animal use followed the NIH guidelines for housing and care of laboratory animals and the guidelines of the Animal Care and Use Committees of the University of Massachusetts Medical School, University of Colorado, and The Jackson Laboratory.

### HIV infection of hu-mice

Humanized mice (hu-mice) were inoculated with infectious HIV NL4-3 (HIV-1_NL-D,_ kindly provided by Dr. Tsunetsugu-Yokota, National Institute of Infectious Diseases, Tokyo, Japan (78–80)) at 3,000 infectious units (IU)/mL (37) diluted in USP-grade 1X-PBS, by tail vein or retro-orbital injection in a final volume of 100 uμl. All mice in this study remained antiretroviral-drug naïve throughout the course of the study.

### Induction of experimental pulmonary arterial hypertension

The two-hit SU5416/hypoxia model is a well-described murine model for PH (59–61). We generated a double-hit model in the context of HIV infection by combining HIV with either hypoxia or SU5416. One subset of mice with or without HIV was placed in a hypoxia chamber with 10% oxygen with an automatic air filtration system (Coy Labs) under normobaric conditions, with food and water *ab libitum.* Briefly, the hypoxia chamber fits mice in their cages in a plexiglass chamber that is hermetically closed. The chamber allows for continuous flow of oxygen at measured levels. In this case, oxygen levels were kept at 10% with nitrogen at 90%, as per standard practices to induce experimental hypoxia. The air inside the chamber was filtered *via* an activated carbon/carbolime filtration system to prevent the buildup of gaseous wastes such as carbon dioxide or ammonia over the course of the experiment. The mice were kept inside the hypoxia chamber 24 hours/day for 28-35 days, until sacrifice. The chamber was opened just briefly every week to weigh the mice for their routine weekly checks and to provide fresh caging, bedding, food, and water. Control mice in normoxia were kept in their cages in room air (21% oxygen). A separate group of mice +/- HIV were injected subcutaneously with 20 mg/kg SU5416 (R&D Systems) or vehicle weekly and housed in normoxia, as indicated in the timeline shown in **Figure 1B**.

### Right heart catheterizations

Mice were subjected to right heart catheterization (RHC) followed by euthanasia when the CD4^+^ T cells in the periphery reached 30% of their nadir value, or at the first sign of weight loss, labored breathing changes in posture, or inability to ambulate for food and water. Briefly, mice were anesthetized by intraperitoneal injection with a sublethal dose of ketamine (70 mg/kg)/xylazine (20 mg/kg) mix followed by intubation using an 18G blunt needle and placed on a ventilator (MiniVent, Harvard Apparatus) on a temperature-controlled surgical platform (Kent Scientific). We measured the right ventricular systolic pressures (RVSP) by averaging 15 tracings (**Figure 3A**) using a 1F pressure-volume catheter, as previously described (Millar PVR-1035; Millar Instruments) (81). Given the nature of these RHC as terminal open-chest procedures, mice were not subjected to RHC at baseline.

### Tissue harvesting

Mouse tissues were collected after RHC. The hearts were used to assess RV hypertrophy. Specifically, freshly harvested cardiac right ventricle (RV) was micro-dissected from the left ventricle (LV) together with the septum (S). We used the formula for Fulton index: RV/LV+S, to calculate the weight ratio of RV and (LV+S) (82). The tracheal intubation needle was disconnected from the ventilator and used to lavage the lungs with 1 mL of 1X PBS. After collection of lung lavage fluids, the right lung lobes were tied off at the right bronchus, excised, and preserved for DNA/protein (flash-frozen in liquid nitrogen) or RNA (in RNA-later solution) and placed in -80C for long-term storage. The tracheal intubation needle was also used to inflate the left lung with warm agarose; the left lung was placed in cartridges, let harden in cold PBS, and then placed in 10% formalin for paraffin embedding. Peripheral blood was collected by exsanguination from either the left ventricle or the abdominal aorta. Organ harvesting was performed after exsanguination and tissues were processed for immunohistochemical and nucleic acid and protein assays.

### Histopathology analyses

Formalin-fixed paraffin-embedded mouse lung tissues were processed for heat-induced antigen retrieval using Leica Bond Epitope Retrieval Buffer 2 (EDTA, pH 9.0) for 20 minutes followed by staining on a Leica Bond automated immunostainer. We used the following primary antibodies for immunohistochemical analyses: α-CD3 antibody (Dako #A045229-2), α-CD68 antibody (Abcam #ab199000), α-Cleaved Caspase-3 antibody (Cell Signaling #9661), and α-Ki67 antibody (Abcam #ab16667). Isotype controls were stained using either rabbit IgG (Abcam #ab172730) or mouse IgG1 (Sigma #M5284). IgG was detected using Dako EnVision Plus Anti-Mouse-HRP-Polymer or Anti-Rabbit-HRP-Polymer and visualized using DAB as a substrate (brown). A hematoxylin nuclear counterstain (blue) was applied. Whole slide images were obtained on a 3D Histech Pannoramic SCAN 150 using brightfield and viewed using CaseViewer version 2.3. Immunohistochemical signals for CD3, CD68, caspase 3, and Ki67 were quantified using the AdipoGauge software package (83). The thickness of the tunica media and vessel diameter were measured as described previously by Henriquez, et al. (84). Briefly, manually outlined segmentation was performed for the external and internal perimeter of the H&E-stained pulmonary vessels from mouse lung sections. First, the contour of each object was outlined as a closed polygon using a digital Pen CTE-440 tablet (Wacom; Saitama, Japan). Next, 2D binary ROIs were generated with a custom-made macro written for the Image SXM software program (85). Thereafter, the masks were calibrated using the Image J software (NIH, USA) for final calculation of vessel wall thickness (T). In order to avoid the effect of the vessel size, the thickness was normalized by their own diameter (D) obtaining the T/D ratio.

### Measurements of pro-inflammatory cytokines

The human pro-inflammatory cytokines IFN-γ, IL-1β, IL-2, IL-4, IL-6, IL-8, IL-10, IL_12p70, IL-13, and TNF-α were measured in 150 μL of neat lung lavage fluids by using electrochemiluminescent sulfo-tag labels as implemented in the multi-spot plates in Proinflammatory Human Cytokines Panel I V-plex kits (Meso Scale Discovery). Analyses were done using MESO QuickPlex SQ 120 and Discovery Workbench software (MSD). The expression of Endothelial-Monocyte Activating Polypeptide II (EMAP II) was assessed by Western blot analyses in mouse lung tissue homogenates. Briefly, lung tissues were homogenized in T-PER solution (Thermo Scientific) supplemented with protease inhibitors (cOmplete Protease Inhibitor Cocktail; Sigma). The tissue lysates were analyzed by SDS-gel electrophoresis using AnykD TGX pre-cast gels (Bio-Rad). The following antibodies were used: rabbit polyclonal anti-EMAP II serum (lot D88) (86) or anti-AIMP1/EMAPII/SCYE1 (Bethyl laboratories # A304-896A-M), rabbit anti-Vinculin (Abcam #ab129002), along with HRP-conjugated goat anti-rabbit secondary antibodies. We imaged the membranes by incubation in Super-Signal West-Femto Substrate (Thermo Fisher) for five minutes, followed by a cumulative 3-min exposure time in Azure C300 imager (Azure Biosystems). Densitometry analyses were done using FIJI software (87).

### Gene expression analyses

Cell-associated total RNA was extracted from right lung tissues preserved in RNA *later* solution, followed by homogenization using a handheld tissue homogenizer. Then, the RNA was run through QIAShredder columns followed by extraction using RNeasy Mini Kit (Qiagen). The quality of the extracted RNA was assessed in an R6K ScreenTape system (Agilent Technologies). RNA (100 nanograms) was reverse transcribed using the RT^2^ First Strand Kit and analyzed using the Endothelial Cell Biology PCR array (Qiagen). All transcripts were detected using SYBR Green qPCR Mastermix (Qiagen, Inc). Transcripts were normalized to housekeeping genes on manual selection of the arithmetic means of ACTB, B2M, GAPDH, and RPLP0. Expression changes were determined using the 2^-ΔΔCt^ method. Datasets were subjected to overrepresentation analyses using Reactome for a better understanding of signaling pathways involved when HIV is combined with hypoxia as a vascular insult.

### Statistical analyses

Data are provided as means ± SEMs. Statistical significance was determined by using the 2-tailed Student *t-*test, ANOVA, the non-parametric Mann Whitney test, or other non-linear regression as appropriate, with corrections for multiple comparisons. Survival curves were analyzed using Kaplan Meier with a Mantel-Cox (log-rank) test. Statistical analysis of the data was performed using GraphPad Prism 8.4.1 software and SPSS Statistics 23.

### Biohazard considerations

HIV-infected hu-mice are fully infectious and represent an important occupational biohazard. All the necessary precautions to prevent bites and scratches to the operator, and/or escaping of mice were always taken. This included sedation of the mice with isoflurane, maximized containment by using benchtop plexiglass enclosures and door barriers, use of long, rubber-tipped forceps to transfer mice between cages, dedicated mouse ventilation and RHC equipment, and extensive use of hospital-grade detergents to clean all work areas and reusable utensils (e.g., Virex or Cavi-wipes). We strived to limit mechanical restraint as much as possible to prevent unnecessary stress on the mice.

## Results

### HIV infection increases inflammatory cytokines in the lungs of humanized mice

**Figure 2:**
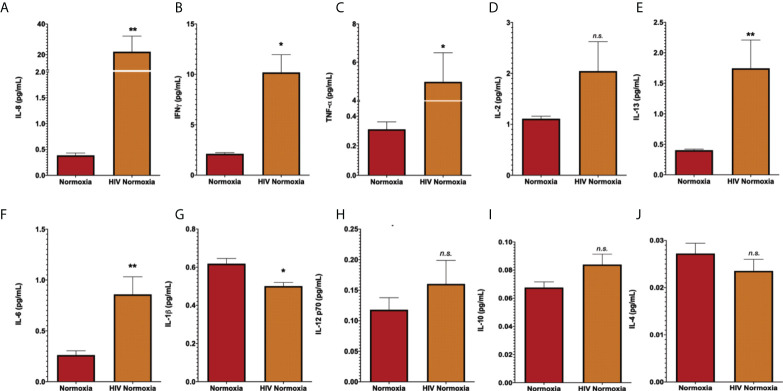
Increased of inflammatory cytokines in lung lavages of HIV-infected humanized mice. Human pro-inflammatory cytokines were measured in lung lavage fluids of humanized mice by electrochemiluminescence, using the MSD Human Pro-inflammatory Panel I 10-plex (Meso Scale Discovery). Panels show detection for IL-8 **(A)**, IFN-a **(B)**, TNF-a **(C)**, IL-2 **(D)**, IL-13 **(E)**, IL-6 **(F)**, IL-1b **(G)**, IL-12 p70 **(H),** IL-10 **(I)**, and IL-4 **(J)**. Neat lavage fluids were diluted 1:200, in duplicates. All values with >20% coefficient of variability were excluded. Data are shown as mean cytokine concentration (pg/mL), SEM; n= 18 mice (5 males and 13 females). Statistical differences (*p < 0.05, **p < 0.01) are indicated by Mann-Whitney tests. ns, non-significant.

The inflammatory response is a common effect between HIV infection and PH. HIV infection causes inflammatory abnormalities in the lungs (88). Inflammation also plays a key role in PH (11), as inflammatory changes occur in the lungs of patients with PH and experimental animal models (65, 89–91). Inflammatory cytokines such as IL-13, IL-6, IL-8, and TNF-α are well-established offenders in PH and predictors of survival in PH patients (92), despite some incongruencies among experimental datasets (93). We were interested in investigating the levels of inflammatory cytokines in neat lung lavage fluids of our hu-mice using electrochemiluminescence. We found that HIV infection significantly increased the expression and release of human IL-8, IL-6, IL-13 (p < 0.01), IFN-γ, TNF- α (p < 0.05), with significant reductions in IL-1β (p < 0.05) in the lungs of HIV-infected hu-mice, compared to uninfected controls **(** **Figure 2** **).** These results confirm that our hu-mice secreted pro-inflammatory cytokines in their lungs upon HIV infection.

### The combination of HIV with either hypoxia or SU5416 promotes PH in humanized mice

**Figure 3:**
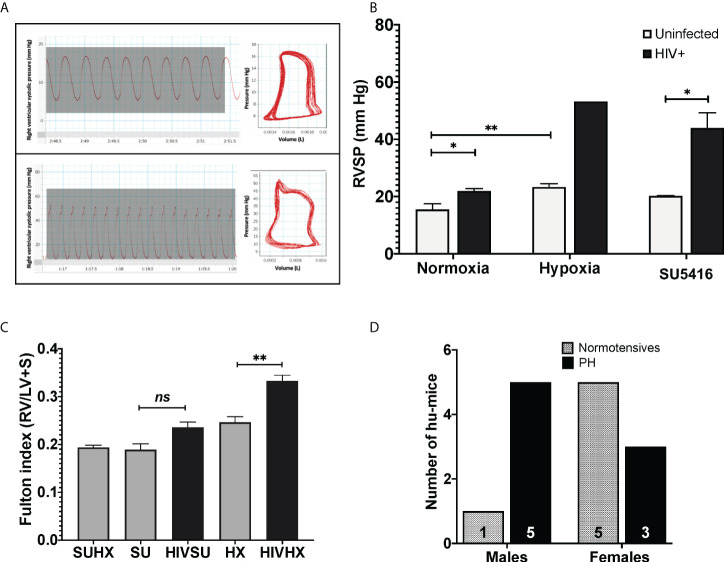
**(A)** Pressure-volume loops and right ventricular pressure tracings in humanized mice infected with HIV. Mice undergoing RHC were anesthetized and placed in a ventilator, with an intra-tracheal canulae. Right ventricular systolic pressures (RVSP) were measured on an open chest, using a 1 femto pressure-volume catheter. For objective measurements, the mean RVSP for each animal was obtained by calculating the average of 15 tracings. PAH designations were based on mean RVSP >25 mm Hg. **(B)** Effects of HIV combined with hypoxia or SU5416 in right ventricular systolic pressure of BLT and CD4+ hu-mice. The effect of the presence of HIV, hypoxia, and SU5416 was evaluated by measuring the RVSP; n=22 mice (10 males, 12 females). Data are shown as mean RVSP +/- SEM. Statistical differences (*P < 0.05, **P < 0.01) are indicated by unpaired T test analyses. **(C)** Right ventricular hypertrophy in humanized mice. Mice subjected to experimental PH with HIV + SU5416 showedright ventricular hypertrophy, as assessed by the Fulton method (RV/LV+S); n=15 mice (5 males and 10 females). Statistical differences (ns, non-significant, **P < 0.01) are indicated by unpaired T test analyses. **(D)** Male-dominant HIV-PH in humanized mice. We analyzed potential sex differences in the subset of mice that were subjected to experimental PH conditions (either hypoxia or SU5416 combined with HIV or SU5416 with hypoxia). Results show that 37.5% of female humanized mice developed PH, compared to 83% males; n=14 mice total.

Mice exposed to hypoxia and SU5416 are well-established models of pulmonary hypertension; however, such an approach has not been tested in HIV-infected mice with humanized immune systems. To this end, we combined either hypoxia or SU5416 in NSG-BLT or NSG-CD34^+^ hu-mice infected with HIV-1 NL4-3 (78–80) **(** **Figure 1** **)**. After a two-week post-infection period, mice were randomly placed in hypoxia (10% O_2_) or kept in normoxia and monitored daily for changes in activity, feeding, or labored breathing and weekly for body weight. Right ventricular systolic pressures (RVSP) were measured by right heart catheterization. We found that HIV alone significantly increased the RVSP of mice in normoxia (p < 0.05, **Figures 3A, B**) and even more when combined with SU5416 (p < 0.05) or hypoxia. Despite monitoring twice a day, HIV+ mice challenged with hypoxia exhibited rapid weight loss and wasting, which made measurements of RVSP unattainable in most mice because they did not survive the pre-RHC steps. For this reason, we were able to measure RVSP in a single mouse, which showed increased pressure (53.2 mm Hg, n = 1). Consistent with other murine experimental models of PH, weekly treatments with SU5416 did not increase RVSP compared to uninfected/normoxic mice. However, hypoxia alone promoted a significant increase in RVSP in uninfected BLT mice (p < 0.01). This observation is consistent with studies from Hu et al. demonstrating that hypoxia increased hemodynamic parameters in humanized mice (75).

Sex differences in PH have been a matter of study for years (97–100). PH shows as a female-dominant disease affecting 4 females per male depending on the etiology of PH. Intrigued by this, we investigated potential trends associated with sex by analyzing the specific datasets of 14 HIV-infected humanized mice that were subjected to experimental PH conditions by treating them with either SU5416 or maintained in hypoxia (6 males and 8 females). Our results suggest PH as an outcome in 1.7 males per female (p = 0.0864, Chi-square test), (**Figure 3D** **),** which suggests a trend of HIV-PH as a male-dominant condition in our mice. This is consistent with our previous studies in HIV-PH patients, in which the majority of PH patients were men (101, 102), as well as in separate registries of patients with HIV-associated PH (103–107).

**Figure 4:**
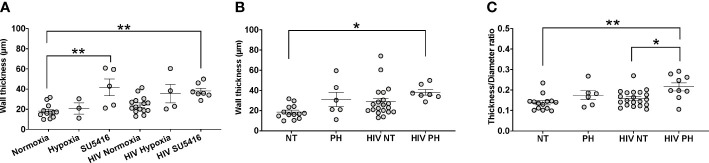
Analyses of tunica media thickness in humanized mice. The thickness of the tunica media and vessel diameter were measured using hematoxylin-stained lung tissue images. **(A)** The wall thickness data analyzed by hit (hypoxia, SU5416, or HIV); **(B)** The wall thickness of different mice models normotensive or pulmonary hypertensive and HIV +/-; **(C)** The ratio of the thickness:diameter comparing different mice models. Each circle represents a vessel in a series of images from different mice (n=2-3 per group). Total number of mice: 22 (7 males and 15 females). Statistical significance is indicated as follows: *, p<0.05; **, p<0.01. Data are depicted as mean, SEM.

One of the histological hallmarks of PH is smooth muscle hypertrophy, which results in decreased vessel compliance and vasoconstriction. Mouse lung sections stained with H&E were used to measure the thickness of the tunica media in vessels measuring 166 ± 9 μm (mean ± SEM) in diameter, which corresponds to small intrapulmonary arteries (108). We compared the tunica media thickness in our HIV-PH mice *versus* mice in normoxia and found a statistically significant increase in the arterial thickness in HIV-infected and uninfected mice treated with SU5416 (p < 0.001, **Figure 4A**). HIV-infected mice in hypoxia tended towards increased wall thickness, albeit non-statistically significant (p = 0.06). When analyzed by phenotype, HIV-infected mice with PH exhibited a significant increase in tunica media thickness, compared to uninfected, normotensive animals (p < 0.01, **Figure 4B**). Because increased thickness may imply a bigger diameter of the vessels, we also analyzed the thickness/diameter ratio (T/D R) in uninfected and infected mice with and without PH. Our findings show that HIV-infected mice had statistically significant increased T/D R compared to uninfected mice in normoxia (p < 0.001) and HIV+ normotensive counterparts (p < 0.01, **Figure 4C**). Altogether, the increased thickness in the pulmonary vascular tunica media observed confirms that the HIV-PH phenotype was achieved in the hu-mice exposed to either hypoxia or SU5416.

**Figure 5:**
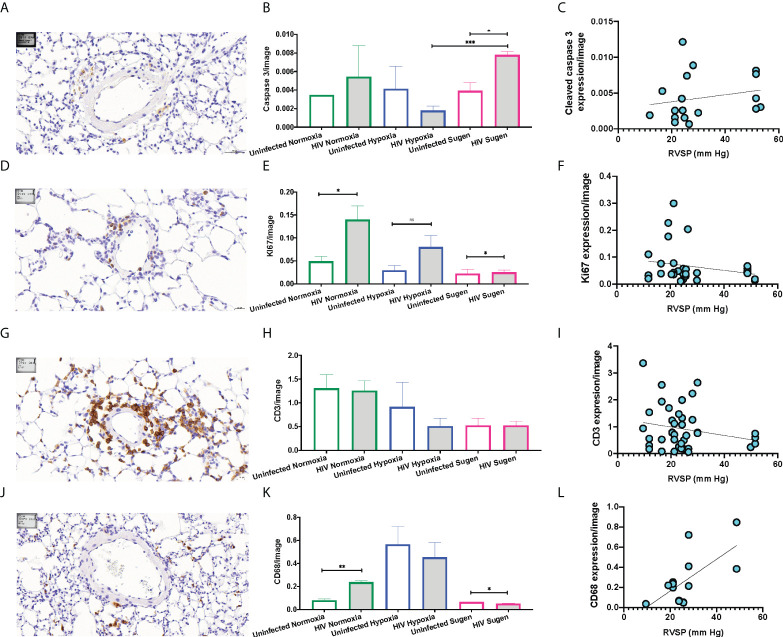
Representative immunohistochemical staining of human cell markers in humanized mouse pulmonary vasculature. Formalin-fixed paraffin-embedded mouse lung tissues were processed and stained on a Leica Bond automated immunostainer using α-Cleaved Caspase-3 **(A)**, α-Ki67 antibody **(D)**, human α-CD3 **(G)**, human α-CD68 **(J)**, primary antibodies with relevant HRP secondary antibodies and isotype controls. Tissues were counterstained with hematoxylin nuclear stain (blue). DAB substrate was applied for detection and the brown signals were quantified and plot in bar graphs for each marker (Panels **B, E, H,** and **K**). The Pearson R squared between the mean Pulmonary Artery Pressures (mPAP) and immunohistochemical staining was calculated using GraphPad Prism (Panels **C, F, I,** and **L**) using data collected from n=33 mice (9 males and 25 females). Statistical significance is indicated as follows: ns, non-significant; *,p<0.05; **p<0.01; ***p<0.001. Data are depicted as mean, SEM.

Immunohistochemical (IHC) staining of lung tissue sections showed that HIV infection trended to increase the expression of active caspase 3 in the mouse lungs at room air (**Figure 5A**). However, HIV+ mice treated with SU5416 showed statistically significant increased caspase 3 expression, compared to their HIV- counterparts (p < 0.01) and HIV+ mice in hypoxia (p < 0.001, **Figure 5B**). There was no significant correlation between the caspase 3 immunostaining and Pulmonary Artery Pressures (mPAP) (R^2^ = 0.04, **Figure 5C**). HIV infection significantly increased the expression of the proliferation antigen KI67 in normoxia (p < 0.01), but not in hypoxia nor with SU5416 treatments (**Figures 5D, E**). There was no significant correlation between the KI67 immunostaining and mPAP (R^2^ = 0.06, **Figure 5F**). The expression of the human lymphocyte marker CD3 was significantly increased in HIV+ mice in normoxia (p < 0.001) but decreased in the presence of SU5416 (p < 0.01, **Figures 5G, H** and **Figure S1**). We found no correlation between the human CD3 expression and mPAP (R^2^ = 0.04, **Figure 5I**). The expression of the human macrophage marker CD68 was significantly increased in HIV+ mice in normoxia (p < 0.001) but decreased in the presence of SU5416 (p < 0.01, **Figures 5J, K**). We found a significant correlation between the expression of CD68 by IHC and mPAP (R^2^ = 0.44, **Figure 5L**), which is consistent with published studies (109–111).

**Figure 6:**
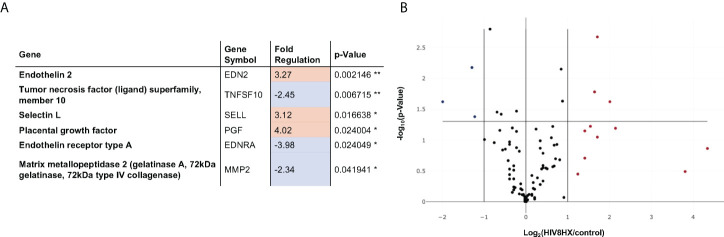
Changes in endothelial cell biology gene expression in HIV-infected hu-mice living under hypoxia, compared to hu-mice in normoxia. Gene expression in the lungs was analyzed using a PCR array targeted to 84 genes relevant in endothelial cell biology. All transcripts were detected using SYBR Green qPCR Mastermix (Qiagen, Inc). Changes in gene expression were determined using the 2^-ΔΔCt^ method in datasets generated from n=12 mice (4 males and 8 females). Panel **(A)** lists six out of 84 genes that had fold changes > 2. The p values are calculated based on a Student’s t-test of the replicate 2^(- Delta CT) values for each gene in the control group and treatment groups, and p values less than 0.05 are indicated with a *, p values less than 0.01 are indicated with **. The p-value calculation used is based on parametric, unpaired, two-sample equal variance, two-tailed distribution. Panel **(B)** shows a Volcano Plot that indicates the level of significance of gene expression relative to the whole 84-gene PCR array.

We next sought to investigate the impact of the combination of HIV + hypoxia in hu-mice on the expression of genes relevant to vascular cell biology, relative to normoxic uninfected controls. Out of 84 genes analyzed, we found that genes associated with vasoconstriction (endothelin-2 and endothelin receptor-A), cell adhesion (selectin L), apoptosis (TRAIL), and angiogenesis (matrix metallopeptidase 2, placental growth factor) were differentially regulated, which is consistent with PH. (**Figure 6**).

**Figure 7:**
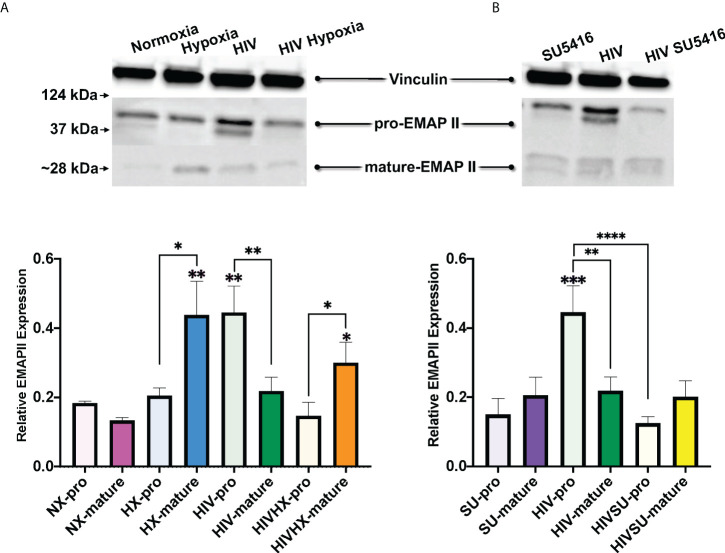
Expression of EMAP II in the lungs of HIV-infected mice with humanized immune systems. Blots show bands for housekeeping protein vinculin (124 kDa), pro-EMAP II at 37 kDa and mature-EMAP II at ~28 kDa, as detected by Western blot. Bars show EMAP II expression normalized to vinculin. Panel **(A)** shows EMAP II dataset comparisons relevant to hypoxia; n=24 mice (5 males, 19 females). Panel **(B)** shows comparisons relevant to mice treated with SU5416 (Sugen); n=24 mice (4 males, 20 females). Mice in normoxia are shown in pink (NX, n=3); hypoxia in blue (HX, n=3); HIV in green (HIV, n=4); HIV + Hypoxia in orange (HIVHx, n=4); SU5416 in purple (SU, n=4) and HIV + SU5416 in yellow (HIVSU, n=4). Pro-EMAP II is depicted in light colors; darker colors show mature EMAP II. Densitometry analyses were performed using FIJI. Data are represented as mean; SEM. Statistical significance was tested using independent T tests using the Fisher’s LSD without corrections for multiple comparisons as implemented in GraphPad Prism 8. Statistical significance is indicated as follows: *,p<0.05; **p<0.01; ***p<0.001; ****p<0.0001.

Endothelial-Monocyte Activating Polypeptide II (EMAP II) has been associated with the progression of HIV and PH. EMAP II is linked to pro-apoptotic and pro-inflammatory pathologies by inducing apoptosis and autophagy in vascular endothelial cells as well as endothelial and monocyte activation. In mouse models, EMAP II is necessary and sufficient for the development of emphysema (112, 113); it also has established roles in HIV-associated pulmonary vascular disease, and in endothelial cell death and inflammation, which are three key players in PH (112, 114–116). Based on this, we were interested in measuring EMAP II in whole lung tissues of our mice challenged with HIV, hypoxia, SU5416, or their combinations. Within the control mice in normoxia, our results showed no differences in the expression of the pro-form and mature forms of EMAP II **(** **Figure 7** **)**. When infected with HIV, we observed an increase in pro-EMAP II expression compared to either normoxia (p = 0.0032) or SU5416-treated mice (p = 0.0001). We also noticed an overrepresentation of the EMAP II pro-form (p = 0.0052) in HIV infection. When analyzed expression of EMAP II in hypoxia as challenge **(** **Figure 7A** **)**. This showed an increase in mature EMAP II compared to normoxia (p = 0.0016), with a significant predominance of the mature form (p = 0.0114). The combination of HIV and hypoxia (HIV-HX) increased mature EMAP II, relative to its pro-form (p = 0.046). However, combinations of HIV and SU5416 (HIV-SU) did not change EMAP II expression in either form **(** **Figure 7B** **)**. HIV-SU significantly decreased pro-EMAP II compared to HIV infection alone (p < 0.0001).

### Graft vs. host disease (GvHD) has a distinctive pulmonary pathology if presented in combination with HIV-PH

A shortcoming with the hu-BLT model is the development of what appears to be chronic xenogeneic graft versus host disease (cGvHD), in which any residual murine major histocompatibility complex (MHC) class I and II antigens are targeted by the human T cells after engraftment (117–120). Several groups of investigators have reported that hu-BLT mice develop this lethal form of cGvHD over several months (cGvHD) (76, 117, 121). Unlike the acute form of xenogeneic GVHD that develops within a few weeks following HSC transplantation that involves the gut, liver, skin, and lung, cGvHD is more protracted and affects skin, liver, eyes, mouth, lungs, gut, salivary glands, and/or neuromuscular system. Greenblatt et al. reported that ~35% of hu-BLT mice develop clinical signs of cGvHD within 5-6 months following HSCs (117). These investigators reported marked infiltration of human T and B cells as well monocytes and macrophages in the skin, lungs, and colon. In another study using hu-BLT mice, Lockridge et al. observed clinical signs of cGvHD at ~100 days post HSC engraftment as well as remarkable immune cell infiltration in the skin, lungs, and live (121). Based on these observations, the development of cGvHD in hu-BLT may be problematic when attempting to dissect the immunopathological mechanism involved in HIV-induced pulmonary disease.

**Figure 8:**
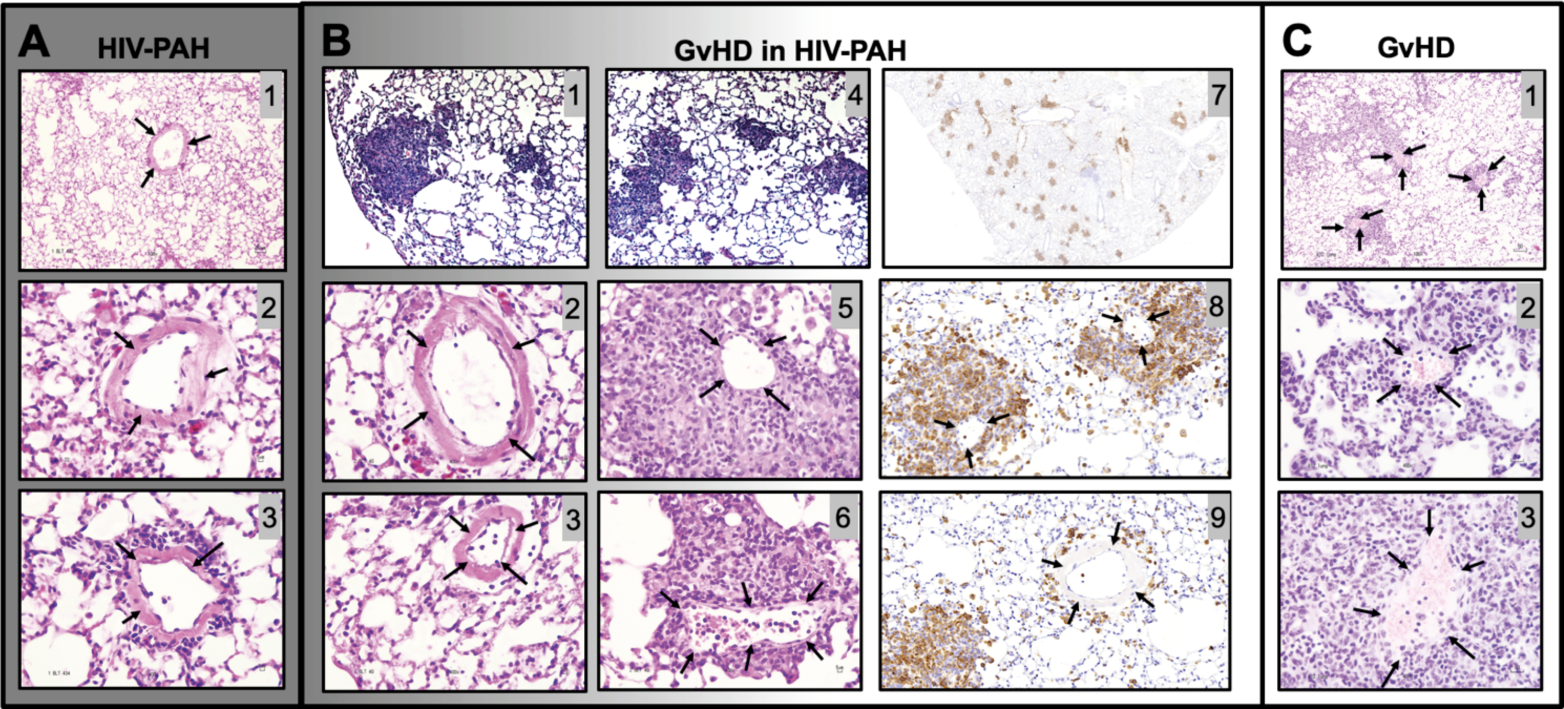
Histopathological comparison between graft vs host disease (GvHD) and HIV-PAH. **(A)** Representative images of formalin-fixed paraffin-embedded lung tissue showing diffused inflammation and muscularized pulmonary vessels in humanized mice with HIV-PH. Mice were exposed to hypoxia and HIV infection. **(B)** Histological characteristics in an HIV-infected humanized mouse that developed GvHD. The mouse presented with a combination of substantial patchy inflammation (photomicrographs 1,4) and diffuse inflammation with evident pulmonary vascular muscularization. Muscularized vessels were outside of the inflammatory patches (photomicrographs 2-3), while significant vascular thinning was observed within the dense inflammatory areas, as shown in photos 5-6. Photomicrographs 7-9 show CD3 immunohistochemistry in the HIV-infected humanized mouse with Pulmonary Hypertension and GvHD. Tissue sections were treated with anti-CD3 antibody, counterstained with hematoxylin (blue), and visualized with DAB (brown). CD3 staining in pulmonary vessels within (Photo 8) and outside (Photo 9) granuloma is shown. **(C)** Classical pulmonary vasculature in a mouse model of allogeneic GvHD presenting muscular thinning within inflammatory patches. Arrows point to pulmonary arteries.

Our mice were monitored daily for the appearance of GvHD symptoms, including weight loss, blepharitis, hunched posture, ruffled fur, hair loss, reduced mobility, and tachypnea. The presence of any of these phenotypes would call for humane euthanasia. In our hands, a single mouse (1 out of 30 mice in this study) presented these symptoms. Because it is conceivable that GvHD may be a confounding factor in PH-associated inflammation leading to increased hemodynamics, we compared the lung histopathology of our HIV-PH mice to that in an established mouse model of GvHD (120) and our mice with HIV-PH without any signs of GvHD. We found diffuse inflammatory infiltrates and muscularized pulmonary vessels in the mice with HIV-PH (**Figures 8A1–3** **)**. Conversely, the HIV-infected mouse that presented the features of GvHD exhibited mixed histopathology with distinct patchy inflammatory infiltrates resembling lung granulomas (**Figures 8B1, B4** **).** The vasculature within the inflammatory patches featured remarkably thinner tunica media **(** **Figures 8B5, B6** **),** compared to the outer areas, which contained thick muscular layers consistent with PH **(** **Figures 8B2, B3** **)**. The prominently thin tunica media were also observed consistently in six mice from a well-established model of allogeneic GvHD (**Figure 8C** **)** (118–120, 122). The perivascular cell infiltrations were identified as CD3+ cells by IHC (**Figures 8B7-B9**). In addition, we investigated the inflammatory cytokine profiles in the lung lavage of these mice using electrochemiluminescence. Our findings, summarized in **Figure S2**, show that mice with HIV-PH have significantly higher expression of IL-8 and IL-1β (p<0.001), as well as a lower expression of IL-12, IL-13 (p<0.05), and IL-14 (p<0.0001) when compared to mice with GvHD. The combination of HIV and GvHD led to statistically significant increases in the expression of IFN-γ, IL-8, and TNF-α (p<0.0001), IL-13, and IL-6 (p<0.01), and IL-2 (p<0.05). There were no significant changes in IL-10, IL-2, or IL-4 in the context of GvHD.

### Increased mortality in hypoxic HIV-infected BLT humanized mice

All mice were observed daily to check on their health condition. Right heart catheterizations followed by humane euthanasia were scheduled at the first sign of disease as indicated above. Statistical comparisons of the median survival using Kaplan Meier with a Mantel-Cox (log-rank) test demonstrate a dramatically decreased survival in HIV+ hypoxic BLT mice compared to HIV+ hypoxic CD34 mice (p=0.0002, **Figure S3**), which affected the performance of RHC on well animals. The HIV-hypoxia mice had a 25% survival rate, while 100% of the HIV- SU5416 mice survived the experimental procedures.

## Discussion

Standard laboratory rodents have modeled pulmonary hypertension when treated with either monocrotaline or Sugen 5416 (SU5416), kept in chronic hypoxia, or treated with combinations of these. Treatment with these chemicals recreate the pulmonary vascular remodeling and hemodynamics changes as observed in human PH (45, 59, 64). The current non-primate models of experimental PH are not susceptible to HIV infection and hence, not suitable for infectious studies in HIV-PH. While chronic infectious HIV disease may not represent an important menace in the era of ART, the damage and repercussions of acute HIV infection in different organs systems remain a threat, at least until the infection gets diagnosed and continued viral suppression is achieved with antiretroviral therapy. Even with complete viral suppression, sporadic bursts in viral replication re-ignite damage induced by infectious HIV, particularly in the vasculature.

HIV transgenic rats and mice are outstanding models at the interface between *in vitro* studies and research with non-human primates or *ex-vivo* human biospecimens. HIV transgenic mice and rats have facilitated thousands of studies, especially within the framework of the impact of HIV proteins in end-organ diseases, all under non-infectious working conditions. PH in itself is overrepresented in people living with HIV regardless of the length of infection, which may be supportive of vascular damage occurring early in the disease. For this reason, the search for a non-primate infectious model of HIV-PH may be warranted. Importantly, such a model could help identify targets for novel therapies to treat HIV-associated PH in people living with HIV.

The humanization of the murine immune system introduced rodent models for use in infectious HIV research several decades ago. Hu et al. (75) demonstrated that immune humanized mice resembled increased RVSP and Fulton index when challenged with hypoxia. Our study not only analyzed additional features but also used humanized mice for the first time in a double-hit approach that combined HIV with either hypoxia or SU5416 to test the suitability of hu-mice for HIV-PH studies.

We found that hypoxia or HIV alone increased RVSP in mice. Furthermore, the combination of HIV infection with hypoxia or SU5416 treatments enhanced inflammation in the pulmonary vasculature, which exacerbated PH in hu-mice. In general, HIV hu-mice co-challenged with hypoxia exhibited significantly increased mortality compared to mice treated with SU5416. One possible explanation for this observation is that hypoxia together with the known function of HIV to induce vascular dysfunction (123) may induce massive oxygen deprivation in the brain and major organs. This high mortality in the HIV-hypoxia group is a major limitation of the hu-mouse model.

Noteworthy, pulmonary vascular obliteration in the form of plexiform lesions, a feature of severe PH in patients, macaques, and rats (51, 53, 57, 124, 125), was not observed in our HIV-PH mouse model. In general, it is well-recognized that mice do not show angio-obliterative lesions nor prominent pulmonary vascular remodeling as well as in rats, larger animals, or humans. Similar to classical rodent models of PH such as hypoxia- or MCT-induced PH, our HIV-PH mouse model recapitulates the first phase of vascular remodeling but not the progressive phase ending with plexiform lesions. In this context, the development of immune-humanized models for HIV-PH that can recapitulate all phases of vascular remodeling would represent an important advancement in the field, given the increasing restrictions concerning research with non-human primates.

This study also described common components in the pathobiology of PH such as increased tunica media thickness, inflammatory cytokines, increased macrophage infiltration into the pulmonary vasculature, as well as expression of EMAP-II. The enhanced EMAP II production through HIV alone is consistent with previous reports showing that HIV envelope protein strongly upregulated EMAP II on the surface of endothelial cells thus causing endothelial apoptosis (126). Given the proinflammatory and TNF-enhancing activities of EMAP II (127), EMAP II production in the vasculature of the infected lung may contribute to inflammation and PH.

The engraftment of mice with human cells/tissues certainly brings inflammatory alterations to the stage, which might serve as confounding factors when studying inflammatory diseases like PH. According to Greenblatt, et al., GvHD is an important issue in BLT mice (116). Our studies observed GvHD in a single animal subject, which was also infected with HIV and treated with SU5416. The animal developed PH but histopathological and inflammatory cytokine quantitative data showed key distinctions between the GvHD and PH pathologies, suggesting a mosaic phenotype in this isolated case. In this context, the newer generations of humanized mice that circumvent GvHD may further advance their application in HIV-PH research (128–131).

This study provides the groundwork for the development of a tractable HIV model suitable for PH studies. Here we focused on HIV not only as the first hit but also as the essential driver of virus-induced vascular injury. Therefore, our model cannot be compared to non-infectious 2-hit models of PH. We conclude that either hypoxia or SU5416 combined with HIV can promote PH in hu-mice. The HIV/SU5416 model may be the most suitable for studies in PH and pulmonary vascular disease due to its significantly higher survival and pulmonary hemodynamics. Our study is limited by the fact that changes in cardiac output were not recorded. Changes in cardiac output may explain why chronic hypoxia led to increased RVSP but not increased vascular remodeling in mice. In addition, it would have been interesting to include mice treated with HIV + SU5416 + Hypoxia but we avoided this approach under the premise that SU5416 + Hypoxia in itself induces PH and that the third hit with HIV would have represented extraneous biological stress to the mice. Nevertheless, this study presents a novel and timely foundation for future infectious studies of HIV-PH using mice, which are considerably more justifiable and tractable models than larger animals. Moving forward, understanding the mechanisms governing the role of hypoxia vs SU5416, as well as BLT vs CD 34 engraftments will help in uncovering the reasons for differences in survival. Recapitulation of angio-obliterative lesions in a humanized rat model challenged with dual hits may also represent a significant refinement to our HIV-PH mouse model.

Altogether, the finding that HIV-infected mice with humanized immune systems and exposed to either hypoxia or treated with SU5416 suggest that a two-hit approach is required for the development of a useful murine model of HIV-PH. It may be useful for further identification of therapeutic targets to treat HIV-associated PH. Future studies may also use the combination with HIV antiretroviral therapy to reflect the fact that most people living with HIV are still at increased risk for cardiovascular comorbidities including PH despite being on antiretroviral therapy.

## Data availability statement

The original contributions presented in the study are included in the article/**Supplementary Material**. Further inquiries can be directed to the corresponding author.

## Ethics statement

The animal study was reviewed and approved by The Institutional Animal Care and Use Committee, University of Colorado Anschutz Medical Campus.

## Author contributions

Conceptualization, SA. Methodology, SA. Validation, AS, ES-M, and FR. Formal analysis, SA, VR-I, AS, JS, FR, MF, and MH. Investigation, VR-I, AS, SA, DA, JS, ES, and ES-M. Resources, SA, BM, FR, NM-M, HM, MG, KP, MF, MH, and MC. Writing—original draft preparation, VR-I. Writing—review and editing, AS, DA, JS, BM, FR, NM-M, HM, MG, KP, ES, ES-M, MF, MH, MC, and SA. Visualization, VR-I, AS, and SA. Supervision, SA. Project administration, SA. Funding acquisition, ES-M and SA. All authors have read and agreed to the published version of the manuscript.

## Funding

This work was supported by the National Heart, Lung, and Blood Institute (NHLBI) award to SA (R21 HL129852) and the National Institute of General Medical Sciences (NIGMS) award to ESM (R25 GM096955) from the National Institutes of Health (NIH).

## Acknowledgments

The authors thank Drs. Michael Brehm and Dale Greiner for assisting with mouse humanization. Edgar G. Martinez, Laura D. Casas, and Ava G. Oliver are acknowledged for their technical assistance, as well as Juliana Fleming for assisting in the animal studies. AS served as Visiting Scholar from the University of Heidelberg.

## Conflict of interest

The authors declare that the research was conducted in the absence of any commercial or financial relationships that could be construed as a potential conflict of interest.

## Publisher’s note

All claims expressed in this article are solely those of the authors and do not necessarily represent those of their affiliated organizations, or those of the publisher, the editors and the reviewers. Any product that may be evaluated in this article, or claim that may be made by its manufacturer, is not guaranteed or endorsed by the publisher.
