# Supplementary material for: Mice with humanized immune system as novel models to study HIV-associated pulmonary hypertension
Source: Front Immunol. 2022 Aug 5;13:936164. doi: 10.3389/fimmu.2022.936164 (PMC9390008; doi:10.3389/fimmu.2022.936164)
Supplement: Supplementary file 1 [file DataSheet_1.docx]

Supplementary Material

**
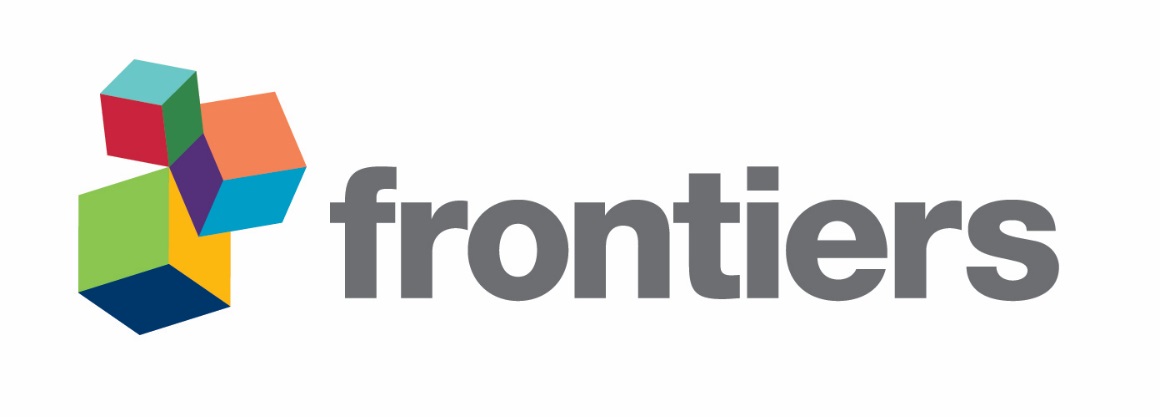
**

**
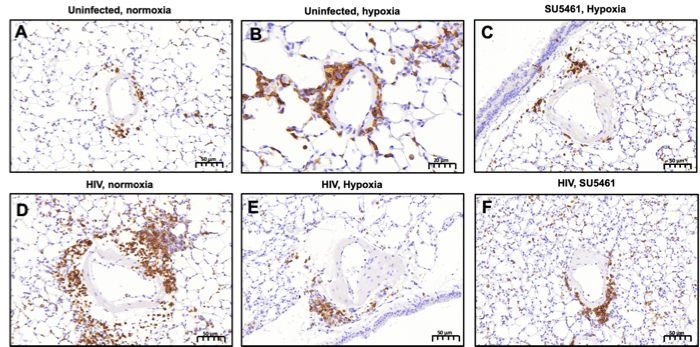
**

**Supplementary Figure 1. CD3 immunohistochemistry in BLT humanized mice.** Representative images of formalin-fixed paraffin-embedded lung tissues of BLT humanized mice. Tissue sections were treated with anti-CD3 antibody, counterstained with hematoxylin (blue), and visualized with DAB (brown). Panel A shows CD3 staining in BLT untreated mouse; Panels B and D show mice exposed to either Hypoxia (B) or HIV (D). Panels C, E, and F show the lung vasculature in the SU6451/hypoxia model (C), HIV/hypoxia (E) and HIV/SU5461 mice. Digital magnifications are provided for each image.


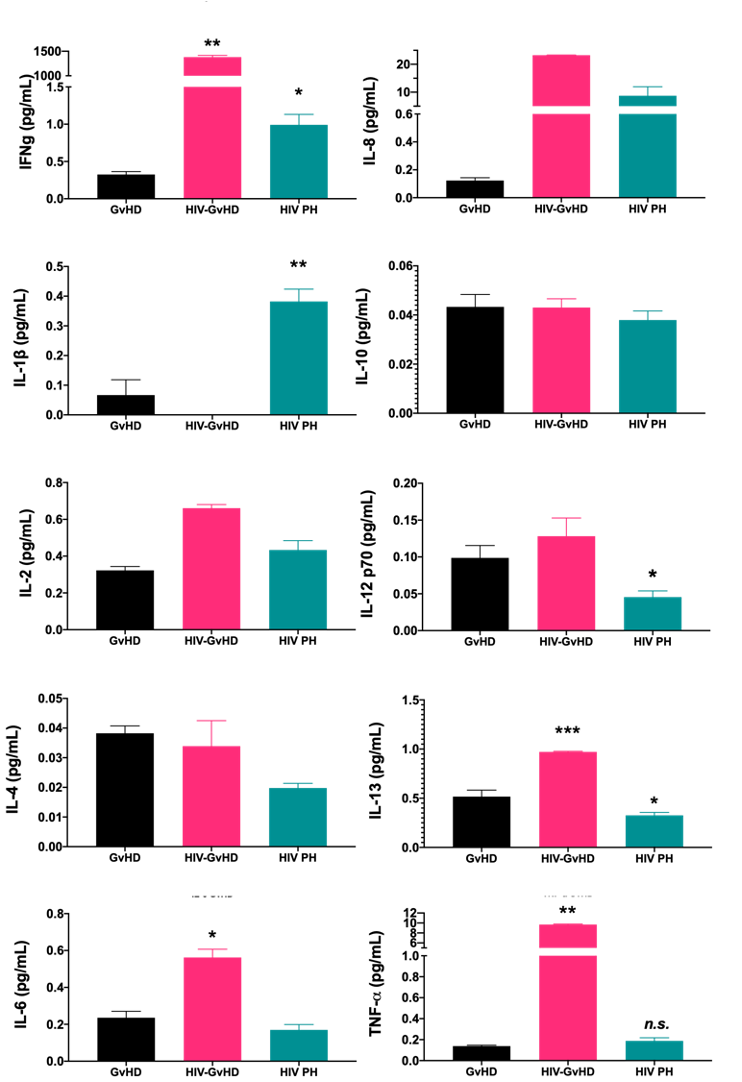


**Supplementary Figure 2. Distinct inflammatory cytokines in lung lavages of HIV-infected humanized mice and mice with GvHD.** Human pro-inflammatory cytokines were measured in lung lavage fluids of humanized mice by electrochemiluminescence, using the MSD Human Pro-inflammatory Panel I (Meso Scale Discovery). Lavage fluids were diluted 1:200, in duplicates. All values with >20% coefficient of variability were excluded. Data are shown as mean cytokine concentration (pg/mL), SEM. Statistical differences (**p* < 0.05, ***p* < 0.01) are indicated by Mann-Whitney tests.

Supplementary Figure 3. Survival analysis of HIV-PH mouse model. Right heart catheterizations followed by humane euthanasia were scheduled when mice showed signs of weight loss, CD4 counts, hunched posture, hair loss, reduced mobility, tachypnea, or inability to ambulate for food and water. Survival time among the HIV-infected mice with pulmonary hypertension were statistically compared using Kaplan Meier with a Mantel-Cox (log rank) test.
